# Multilocation comparison of fruit composition for ‘HoneySweet’, an RNAi based plum pox virus resistant plum

**DOI:** 10.1371/journal.pone.0213993

**Published:** 2019-03-22

**Authors:** Ann M. Callahan, Chris D. Dardick, Ralph Scorza

**Affiliations:** United States Department of Agriculture, Agriculture Research Service, Appalachian Fruit Research Station, Kearneysville, West Virginia, United States of America; Instituto de Biologia Molecular y Celular de Plantas, SPAIN

## Abstract

‘HoneySweet’, a transgenic plum (*Prunus domestica*) resistant to plum pox virus through RNAi, was deregulated in the U.S. in 2011. The compositional study of ‘HoneySweet’ fruit was expanded to include locations outside of the US as well as utilizing a wide variety of comparators and different collection years to see the variability possible. The results revealed that plums have a wide variation in composition and that variation among locations was greater than variation among cultivars. This was also the case for different years at one location. The results supported the supposition that the transgene and insertion event had no significant effect on the composition of ‘HoneySweet’ fruit even under virus pressure, and that it fell in the normal range of composition of commercially grown plums. It also suggested that the effect of environment is as great as that of genetics on the fruit composition of plums.

## Introduction

Compositional analysis is used to demonstrate substantial equivalence for genetically modified (GM) food and feed. If the GM product does not differ in composition from an isogenic line or if its composition falls in the realm of commercial lines, then it is concluded that the product does not have any additional risk relative to the non-GM product [1]. In woody perennial species where transformation of a cultivar is possible, the cultivar with and without the transgene can be compared, the difference between the transgenic and the source clone being only the transgene and the insertion site. In the case where the transformation method is performed on sexual tissues (embryos for example), then the source cultivar is not necessarily a good comparator due to random assortment of genes of both parents. In such cases it would require several generations (4+) to produce near isogenic lines. This is not reasonable in commodities that have long juvenile periods where an inbreeding program to create near isogenic would require decades, nor in polyploid crops where inbreeding depression would preclude the development of isogenic lines [2].

‘HoneySweet’ plum (*Prunus domestica*), a GM product resistant to plum pox virus (PPV) through an RNAi mechanism [3], was developed from transformation of embryonic tissue derived from fertilization of a fresh market plum, ‘Bluebyrd’ by an unknown parent [4–5]. The juvenility period is from 4–10 years for plum and it is hexaploid and highly heterozygous, hence the lack of a near isogenic comparator. In this case the most feasible approach to evaluate the fruit of this transgenic clone is to compare it with a range of commonly available plum cultivars. An initial analysis of fruit composition determined that ‘HoneySweet’ fruit did fall within the range of several commercial plums grown at the same location and the same year [6]. This composition study provided supporting data for the deregulation of and release of ‘HoneySweet’ in the United States [7]. To be able to export or grow ‘HoneySweet’ outside of the United States, a broader look at factors affecting fruit composition was needed, specifically, growing ‘HoneySweet’ in other locations such as Europe in the presence of PPV as well as appropriate comparators and multi-year trials.

In designing this broad look at plum composition, there were several issues that needed to be addressed including which components to analyze, orchard design and orchard management. The choice of components is typically based on OECD consensus documents as well as the Codex Alimentarius which give guidance on the components that are important positive and negative to the use of the product. For plum the OECD consensus document provides no guidance on particular traits of importance [8]. The Codex Alimentarius suggests that “key nutrients and antinutrients be analyzed—defined as those components in a particular food that have a substantial impact in the overall diet” [9].

However, plums are a small part of the daily diet for most people. In the United States the estimated per capita fruit consumption in 2015 was ~186 lbs. of which plums (fresh, dried, juice) are approximately 1.06 lb [10]. At that level, little would have a substantial impact in the overall diet. But, plums are generally thought of as a healthy, nutritious food, even a functional food [11]. Plum fruit is high in polyphenols [12–14] and support good digestive health [15]. Dried plums, in particular, have been shown to protect against and reverse bone loss in rodent models as well as humans [16–22]. The fruit composition of plums has been analyzed with respect to antioxidant and polyphenolic compounds as well as sugars with the idea that those aspects contribute to their special health benefits [23]. We chose to primarily analyze sugars, acids, overall antioxidant and phenolic compounds, vitamin C, various minerals as well as ash and fiber as being the general characteristics of fruit and health associated characteristics.

The second aspect, that of orchard design and management planning in order to minimize confounding factors in an analysis of composition is especially difficult with fruit trees and GM trees as well. The number and location of ‘HoneySweet’ trees was limited by the necessary permits needed to plant a GM tree. Our approach was to choose a wide variety of locations and management plans to obtain the widest array of results.

The overall objective of this study was to analyze plum fruit from a broad range of conditions to determine if ‘HoneySweet’ remained substantially equivalent to plum. ‘HoneySweet’ fruit grown in Europe was included in the analyses to see the effects of PPV, as well as different environments on composition. Comparators, representing the wide range of commercially grown plum, were sampled from 12 locations, including Europe, where they were subjected to PPV pressure. These would give the broadest range of environments and management programs to see the effects on composition for plum.

And lastly fruit from different years grown in one location, to again determine the range of variation possible under same location and management. To this end, ripe plum fruit from 23 cultivars grown in Europe, the U.S. and Canada were analyzed including multiple locations in Europe for ‘Stanley’ a PPV tolerant cultivar, ‘Jojo’ a PPV resistant cultivar and ‘HoneySweet’. In addition, two cultivars grown at one location in the U.S. as well as ‘HoneySweet’ were collected for three different years. These analyses were performed to determine if ‘HoneySweet’ provides a nutritional profile similar to a wide range of plum varieties that are available in the market and therefore would provide all of the health benefits that are generally ascribed to the intake of plums as part of a healthy diet.

## Materials and methods

### Plant materials

Fruit were collected by the scientists in charge of the orchards (see Acknowledgments) from 1 to 4 trees from 12 different orchards, 10 in Europe and 1 each in Canada and the United States. ‘HoneySweet’ grown in the Czech Republic were grown under the Ministry of Environment of the Czech Republic GM planting No. 881/OER/GMO/01 and the field trial was extended, Ministry Reference Number 41538/ENV/09 issued on September 18, 2009. Permissions for field release of GMO Nos. B/ES/96/16 and B/ES/05/14 (‘HoneySweet’) was given by the Spanish Ministerio de Medio Ambiente. In addition, one cultivar, Prunier d’ente, was collected from the market in Bordeaux, France. Orchard design and management was site specific. Fruit (>10) was sampled from each tree at harvest (ripe stage) and shipped under APHIS permit number P526P-11-02618, (or brought in from the field) to Kearneysville, WV where stones and seeds were removed and flesh and skin from each fruit were frozen in liquid N2 and stored at -80 C. The specific cultivars and their location sources are listed in Table 1.

**Table 1:** Cultivars and locations analyzed for fruit composition.

Cultivar	# Trees Sampled	Duplicate Sample^a^	Second Sampling Time^b^	Total # Samples Analyzed	Location
HoneySweet	4	1		5	Czech Rep.
HoneySweet	2	2	2(H)	6	Spain (S)
HoneySweet	>4^c^		3(Y)	3	United States
JoJo	4			4	Czech Rep.
JoJo	2			2	Poland
JoJo	>9^c^		3(Y)	3	United States
Stanley	2			2	Bulgaria
Stanley	1			1	Czech Rep.
Stanley	3			3	Italy
Stanley	1			1	Romania
Stanley	2			2	Serbia
Stanley	3	2		5	Spain (N)
Stanley	2			2	Spain (S)
Stanley	>4^c^		3(Y)	3	United States
Altans Gage	1			1	Bulgaria
Cacanska Najbolja	1			1	Bulgaria
Pacific	1			1	Bulgaria
Blue Bell	1			1	Canada
Italian Prune	1			1	Canada
Vision	1			1	Canada
Prunier D'ente	1			1	France
Haganta	1			1	Germany
Toptaste	1			1	Germany
Helena 10	1			1	Italy
Presenta 7	1			1	Italy
Amers	1			1	Poland
Jubileu 50	1			1	Romania
Romaner	1			1	Romania
Cacanska Rodna	1			1	Serbia
Pozegaca	1			1	Serbia
President	1			1	Spain (N)
Reine Claude de Bavay	1			1	Spain (N)
Reine ClaudeTardive Chambourcy	1			1	Spain (N)
Valor	1			1	Spain (N)

^a^ # of samples reanalyzed

^b^Number of trees that were sampled either at a second harvest time(H) or a different year(Y)

^c^Number of trees sampled and fruit pooled

### Composition analyses

Sixty-two samples, including the duplicates and different harvest times (Table 1), were sent on dry ice in six batches to Medallion labs (Golden Valley, MN) for processing. These samples were shipped under APHIS permit number P526P-12-02899 to Medallion labs because of the potential for the presence of PPV in the European samples. Approximately 150–350 g of each sample was ground, and appropriate amounts were sampled for each assay. The assays for each component analyzed are listed in S1 Table. The following components were measured: starch, carbohydrates, calories, fiber, moisture, ash, antioxidant activity, carotenes, acidity, acids, sugars, sugar alcohols, vitamins, total phenolics, Fe, Ca, Mg, Na, and K. Results were tabulated and analyzed based on amounts per 100g of fresh weight.

### Statistical analyses

Data were analyzed using the Statistical Analysis System version 9.2 (SAS Institute, Cary, NC, USA). An ANOVA, using the general linear procedure (PROC GLM), was performed at α = 0.05 significance level. The sample means were compared using the Tukey-Kramer multiple comparison test (α = 0.05). Graphs were generated using Excel.

## Results

### Fruit sampling and composition

**Fig 1:**
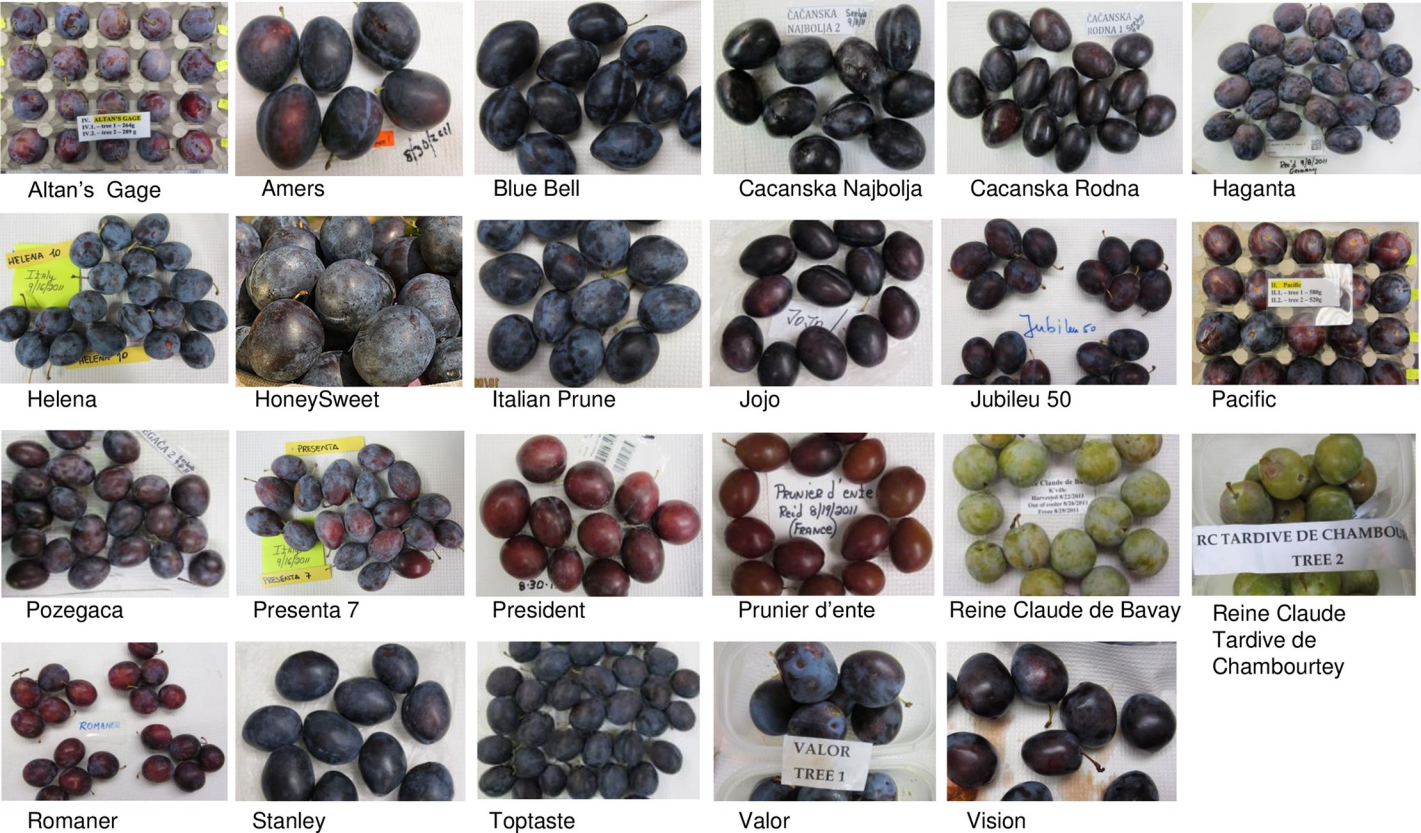
Composite of all the plums used in the composition analyses. Picture of the fruit as it was received. ‘HoneySweet’ picture is from previous fruit.

In order to determine if ‘HoneySweet’ fruit composition was significantly altered by the presence of the transgenes, it was compared to a wide variety of cultivars grown under different environmental conditions including a range of latitudes (39.38–51.95) and longitudes (24.74–79.39) that span the whole northern temperate zone. Fruit from 23 cultivars were collected from 10 locations in Europe, one location in the United States and one location in Canada (CA) (Table 1). All the European locations were subject to pressure from PPV infection. Collections were made in three different years at the United States (US) location and at two different harvest times in the south of Spain (ESs) to enable comparisons in different growing seasons and different harvest times from the same trees. Two of the samples from ESs and two of the samples from Bulgaria (BA) of the cultivar Stanley were found to have been infected with PPV even though the fruit had no detectable symptoms (Callahan, Dardick, Malinowski, Ravelonandro and Scorza personal communication). Most of the collections were made from individual trees with the exception of the samples in the US in which fruit were pooled from multiple trees and the ‘Prunier D’ente’ from France (FA), which came from the commercial fruit market. The fruit had been harvested ripe but still firm and photographed to verify the maturity by color (Fig 1).

The fruit represented a range of diverse plum types including the ‘Green Gage’ ‘Reine Claudes’, large red fruiting types like ‘President’ and ‘Romaner’, small purple ‘Pozegaca’ and ‘TopTaste’ plums, as well as the larger purple plums ‘Stanley’, ‘Vision’ and ‘HoneySweet’. Three of the cultivars were sampled in at least three locations, ‘HoneySweet’, ‘Stanley’ and ‘Jojo’.

**Fig 2:**
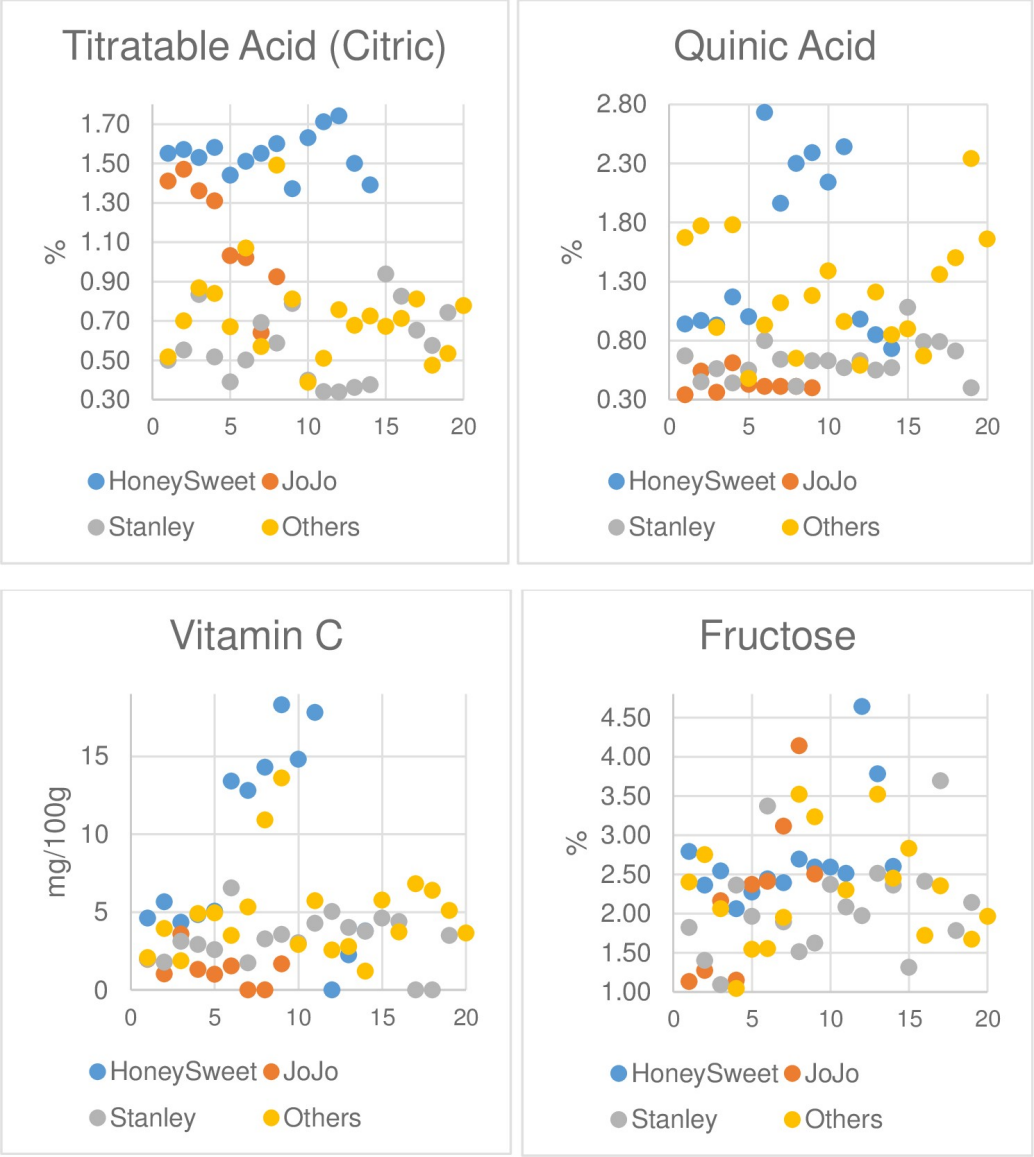
Distribution of values for individual fruit samples for four components. The three multilocation samples, ‘HoneySweet’ in blue, ‘Stanley’ in grey, ‘Jojo’ in orange and the other 20 cultivars in yellow, are graphed for Titratable Acid, Quinic Acid, Vitamin C and Fructose. ‘HoneySweet’ is consistently high for titratable acid, and ‘Stanley’ and ‘Jojo’ are consistently low for quinic acid. All the others overlap.

Fruit was analyzed from a total of 62 plum samples including 14 samples of ‘HoneySweet’, nine samples of ‘Jojo’, 19 samples of ‘Stanley’ and 1 sample from each of 20 other cultivars that are grown in Europe or Canada as well as the United States (Table 1). The 54 components analyzed were chosen based on attributes associated with plum (sugars, acids, antioxidants, sugar alcohols, vitamins, fiber) as well as a known anti-nutrient component [24]. Table 2 presents the averaged results, standard deviation and Tukey-Kramer grouping for each of the three cultivars and the pooled ‘others’ for 30 different components. There were only small differences in the averages of most of the components. ‘HoneySweet’ fell into a unique grouping for higher ash, vitamin C, titratable acidity and malic acid. ‘Stanley’ and ‘Jojo’ deviated from the rest of the plums in having a lower level of quinic acid. Fructose, the major fruit sugar, had very little variations amongst all the plum samples with an average of 2.05% to 2.73%. Fig 2 presents the values from all the individual fruit samples for titratable acid, quinic acid, vitamin C and fructose as examples. The degree of variation between groups as well as within groups demonstrates that there is not a defined amount of any particular component in commercially acceptable plum fruit.

**Table 2:** Average values for composition of plums.

Test	Assay	Unit	Stanley	JoJo	HoneySweet	Others
Carbohydrates	16	%	^a^17.2(0.67)ab^b^	15.2(1.04)b	19.8(1.30)a	18.3(0.61)ab
Calories	16	/100g	72.4(2.92)ab	62.1(4.20)b	84.6(5.86)a	77.6(2.54)a
Protein,by Dumas	15	%	0.94(0.07)a	0.66a	1.25(0.14)a	1.17(0.06)a
Dietary Fiber	14	%	2.44(0.10)b	2.16(0.06)b	2.54(0.16)b	3.57(0.25)a
Moisture	10	%	81.6(0.74)ab	84.1(1.11)a	78.4(1.50)b	80.1(0.65)b
Ash,Overnight	7	%	0.47(0.02)b	0.45(0.05)b	0.61(0.04)a	0.45(0.02)b
Fat,Gravimetric(Total)	11	%	0.16(0.02)a	0.12(0.03)a	0.15(0.02)a	0.02(0.01)b
Calories from Fat	11	/100g	1.42(0.19)a	1.11(0.35)a	1.36(0.23)a	0.20(0.41)b
VitaminC	12	mg/100g	3.53(0.30)b	1.73(0.34)b	9.38(1.63)a	4.88(0.67)b
Iron	13	mg/100g	0.33(0.01)a	0.31(0.02)a	0.31(0.02)a	^c^ND
Niacin	9	mg/100g	0.46(0.04)a	0.34(0.03)a	0.49(0.06)a	0.47(0.05)a
Calcium	13	mg/100g	9.64(0.48)a	8.56(0.84)a	10.9(1.79)a	10.2(0.74)a
Magnesium	13	mg/100g	8.61(0.23)a	6.82(0.14)ab	6.23(0.75)b	7.41(0.46)ab
Sodium	13	mg/100g	22.8(1.02)ab	28.6(2.89)a	21.9(1.54)b	25.0(1.63)ab
Potassium	3	mg/100g	215(11.4)ab	177(7.72)b	241(18.3)a	192(7.64)b
AntioxidantActivity	17	mmoles TE/100g	2084(117.5)ab	1744(100.2)b	2050(219.3)ab	2500(205.8)a
transbetacarotene	6	IU/100g	762(105.5)a	624(87.2)ab	325(55.8)b	536(78.4)ab
cisbetacarotene	6	IU/100g	77.3(9.95)a	51.6(4.69)ab	56.4(7.21)ab	40.8(6.16)b
TotalbetaCarotene	6	IU/100g	779(117.8)a	664(95.5)ab	330(64.9)b	575(83.7)ab
TotalCarotene	6	IU/100g	780(118.3)a	676(97.3)ab	330(64.9)b	581(85.7)ab
Phenolics	18	mg GAE/Kg fw	503(48.5)a	595(82.9)a	532(56.9)a	791(91.0)a
TitratableAcidity(Citric)	4	%	0.57(0.04)c	1.11(0.10)b	1.55(0.03)a	0.73(0.05)c
Malic	5	%	0.94(0.06)c	1.59(0.09)b	1.85(0.06)a	0.71(0.05)c
Quinic	5	%	0.62(0.04)b	0.43(0.03)b	1.54(0.20)a	1.20(0.11)a
Sorbitol	19	%	2.25(0.26)ab	1.56(0.44)b	3.32(0.66)a	3.30(0.29)a
TotalSugarAlcohols	19	%	2.25(0.26)ab	1.56(0.44)b	3.32(0.66)a	3.30(0.29)a
Sucrose	19	%	3.97(0.39)ab	2.70(0.38)bc	2.50(0.26)c	4.42(0.30)a
Fructose	19	%	2.09(0.15)a	2.25(0.33)a	2.73(0.18)a	2.05(0.19)a
Glucose	19	%	4.44(0.15)ab	3.86(0.32)b	5.23(0.33)a	4.10(0.19)b
TotalSugars	19	%	10.49(0.44)a	8.81(0.79)a	10.47(0.47)a	10.58(0.30)a

aThe values indicate average (standard error of the mean) Tukey-Kramer grouping for N trees (N = 19 for Stanley, N = 9 for JoJo, N = 14 for HoneySweet, N = 20 for others).

^b^Different letters indicate a significant difference in composition (Tukey-Kramer test, α = 0.05).

^c^ND indicates no data available.

S2 Table and S3 Table present a summary of the measurements of 10 components for which less than half of the fruit had amounts above the minimal detection limits and a list of 14 components that no fruit had levels at or above the limits of detection. For example, only nine of the 62 samples had detectable values for alphacarotene, for which 17 cultivars including ‘HoneySweet’ samples had below the minimal detectable level. For the anti-nutrient component oxalic acid, seven cultivars including ‘HoneySweet’, had at or above minimal detectable levels. The level detected though was relatively low, equivalent to that normally found in sweet corn and kale [25].

### Variation by location

**Fig 3:**
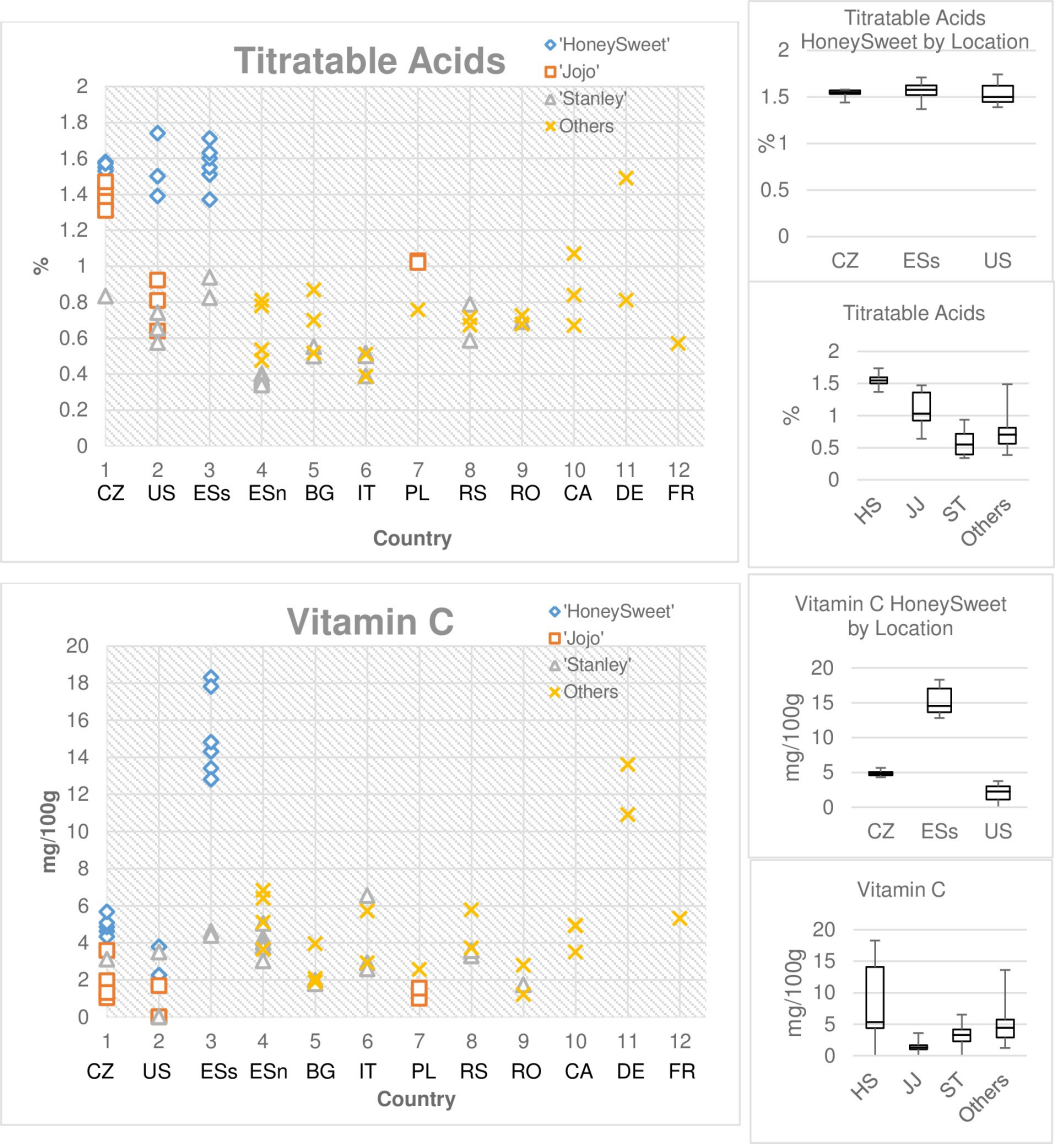
Effects by location. Titratable acid is again consistently high in ‘HoneySweet’ regardless of the location. In contrast, the vitamin C level is affected by location. Where it is consistently high in the southern Spain location for ‘HoneySweet’ as well as two cultivars in Germany. The box plot graphes further show the effect using averages and high and low points. CZ-Czech Republic, US-United States, ESs-southern Spain, ESn-northern Spain, BG-Bulgaria, IT-Italy, PL-Poland, RS-Serbia, RO-Romania, CA-Canada, DE-Germany, FR-France, HS-‘HoneySweet’, JJ-‘Jojo’, ST-‘Stanley’.

To determine what effect location had on the composition of the fruit, a similar analysis was done comparing cultivar by location (S3 Table). Strikingly, ‘Stanley’ had no significant differences in composition by location. The Tukey-Kramer groupings overlapped for each of the components. ‘Jojo’ on the other hand varied by location for several of the components including sugars being lower and acids being higher in the Czech Republic and carotenes being lower and sorbitol being higher in the United States. ‘HoneySweet’ also varied by location, with ESs having higher values for dietary fiber, antioxidant activity, ash, potassium, carotenes, quinic acid, sorbitol and vitamin C. US fruit having lower levels of glucose but higher levels of sucrose. In looking at the ‘others’ class of 20 cultivars at nine locations only one component, vitamin C was significantly higher in Germany. The individual measurements separated by geographical location were graphed (S1 Fig). Two examples are shown in more detail (Fig 3). The levels of titratable acid still appeared to be consistently higher in all the ‘HoneySweet’ samples. There were five other samples that had similarly high titratable acid, the four ‘Jojo’ samples from the Czech Republic (CZ) and the single ‘Haganta’ sample from Germany (DE). The commercial cultivar ‘Bluebyrd’, the maternal parent of ‘HoneySweet’, was also found to have a high titratable acid (6). Titratable acid levels in ‘Jojo’ appear to show a location effect as the samples from the CZ are all high relative to those from Poland (PL) or the US.

In contrast, the high levels of vitamin C found in ‘HoneySweet’ appear to show a location specific effect (Fig 3) rather than an overall high level. Distinctly higher levels were only found in ‘HoneySweet’ grown in ESs, levels two to three times higher than the other ‘HoneySweet’. Only two other cultivar samples, both from DE, had similar high levels. The remaining cultivars had levels 33 to 50% of those seen in the ESs samples. Similar to variations in vitamin C observed in ‘HoneySweet’, variation in various components in ‘Jojo’ and ‘Stanley’ also correlated with location (S1 Fig). The levels of phenolics and total carotenes, both important components for potential antioxidant activity, exhibited two- to seven-fold differences by location. Total phenolic content (mg GAE/Kg fw) in the three plum cultivars ranged from 354 to 862. ‘HoneySweet’ had average values of ~373 in CZ, 706 in ESs and 450 in the US, while ‘Jojo’ had average values of ~755 in CZ, 609 in PL and 372 in the US and ‘Stanley’ ranged from a low average of 354 in BA to a high of 862 in Romania (RA). Total carotene (IU/100g) ranged from 76 to 923. ‘HoneySweet’ had average values of 169 in CZ, 591 in ESs and only 76 in US. ‘Jojo’ had 828 in CZ, 908 in PL and 317 in US. ‘Stanley’ had a low of 155 in US and a high of 923 in CZ. Individual graphs showing the ranges for components with variation are shown in S1 Fig.

### Variation by batch, collection time, and harvest year

To measure compositional variation of fruit from the same tree, three approaches were taken. The first was to test the variation in sampling by analyzing a second sample from the same batched fruit. This would measure the technical variation as well as any variation in the uniformity of the fruit sampled in one batch. There was a very close association of many of the values (Table 3, S4 Table), in particular the overall sugar, moisture, titratable acid, antioxidant activity and vitamin C contents. This suggested that the technical variation is low as well as that the batching was uniform, and variations seen were not just the result of non-representative batching.

**Table 3:** Subset of composition components of duplicate samples and multiple collection times.

Sample Name	Duplicate	Country	Cultivar	Calories	Titratable	Antioxidant	Moisture	Total	VitaminC
				/100g	Acidity %	Activity μmoles TE/100g	%	Sugars%	mg/100g
CZ HS V/2	Duplicate^a^	Czech Rep.	HoneySweet	64	1.6	1300	83.8	9.1	4.6
CZ HS V/2	Duplicate	Czech Rep.	HoneySweet	64	1.6	1300	83.6	8.7	5.7
ES HS 5.3 7/25	Duplicate	Spain (S)	HoneySweet	105	1.6	2300	73.0	11.7	12.8
ES HS 5.3 7/25	Duplicate	Spain (S)	HoneySweet	108	1.6	2700	72.7	11.4	14.3
ES HS 5.3 7/21	Date^b^	Spain (S)	HoneySweet	101	1.5	2500	74.4	11.6	13.4
ES HS 7.3 7/25	Duplicate	Spain (S)	HoneySweet	107	1.6	2700	72.7	11.9	14.8
ES HS 7.3 7/25	Duplicate	Spain (S)	HoneySweet	112	1.7	3100	71.5	12.0	17.8
ES HS 7.3 7/21	Date	Spain (S)	HoneySweet	111	1.4	3500	71.4	12.6	18.3
ES II ST T1	Duplicate	Spain (N)	Stanley	87	0.4	2100	78.1	13.1	4.0
ES II ST T1	Duplicate	Spain (N)	Stanley	86	0.4	2000	78.0	12.8	3.8
ES II ST T 2	Duplicate	Spain (N)	Stanley	88	0.3	1800	77.8	13.5	4.3
ES II ST T 2	Duplicate	Spain (N)	Stanley	87	0.3	1800	77.9	13.0	5.0
US HS 2008	Year^c^	United States	HoneySweet	87	1.7	2700	77.9	12.6	ND^d^
US HS 2010	Year	United States	HoneySweet	79	1.5	1500	79.9	11.1	2.2
US HS 2011	Year	United States	HoneySweet	56	1.4	1000	85.5	7.3	3.8
US ST 2008	Year	United States	Stanley	65	0.6	1800	83.6	9.9	ND
US ST 2010	Year	United States	Stanley	82	0.7	1700	79.3	10.8	ND
US ST 2011	Year	United States	Stanley	57	0.7	1700	85.4	8.2	3.5
US JJ 2008	Year	United States	JoJo	80	0.6	1300	79.3	12.0	ND
US JJ 2010	Year	United States	JoJo	75	0.9	1300	80.8	10.4	ND
US JJ 2011	Year	United States	JoJo	68	0.8	2100	82.5	9.9	1.7

^a^Duplicate—a second sample was analyzed from the same tree at the same time.

^b^Date—a sample was collected on a second date, 4 days earlier.

^c^Year—similar sample was collected in a different year

^d^ND—amount was below the minimal detection level for the assay.

The effect of the collection time on variation was also monitored. Two ‘Stanley’ trees in north of Spain (ESn) were sampled twice, four days apart (Table 3, S4 Table). Those values as well as a duplicate of the later sampling date have some variability as 8 of the 60 values deviated by 10–14% (Table S4) while fat had a variation in one of the ‘Stanley’ samples of 26% and of 18% in sodium in the other ‘Stanley’ sample These small variations suggest that the larger variations are not primarily due to the time of sampling fruit.

Fruit was sampled from the same trees for three different years for ‘HoneySweet’, ‘Jojo’, and ‘Stanley’ in Kearneysville, WV (US). This would be sampling different environmental times for the same trees managed in the same manner. In these samplings larger variations were seen. ‘HoneySweet’ and ‘Jojo’ exhibited large variation, in calories (30%), antioxidant activity (80%), moisture and total sugar (34%) among others (Table 3, S4 Table). For ‘HoneySweet’ the antioxidant activity ranged from 1000 to 2700 depending on the year, while for ‘Jojo’; it ranged from 1300 to 2100 and for ‘Stanley’ it was nearly the same for all three years. ‘Stanley’ showed smaller variations but, sugars and sorbitol varied by year (S4 Table). The effect of the year greatly influenced the variation unlike repeat samplings from the same tree in the same year.

### Variation by PPV infection

Lastly, there have been reports in the literature regarding the effects of PPV infection on fruit quality, primarily on antioxidants as well as sugars [26–27]. ‘Stanley’ is known as a PPV-tolerant cultivar such that when it is infected it exhibist leaf symptoms but no fruit symptoms, allowing fruit to be marketed [28]. Here we analyzed 4 samples from infected ‘Stanley’, two from Bulgaria (BG) and two from ESs. These were compared to 8 samples of ‘Stanley’ that were not infected, 5 from ESn and 3 from the US. The analysis showed very little variation among ‘Stanley’,which could be associated with the presence of PPV. ANOVA analysis indicated two components, carbohydrates and total sugar, had a significant ρ <0.05 (S4 Table).

Conversely, the potential pressure of the presence of PPV containing aphids for numerous years was analyzed in ‘HoneySweet’, comparing 8 samples from Europe with the three samples from US. A number of components had a significant ρ <0.05(S4 Table). In this case the trees in Europe had higher carotenes, sucrose levels and vitamin C but lower fructose.

## Discussion

Biotechnology risk assessment typically includes compositional studies of transgenic and non-transgenic counterparts to determine if the presence of transgenes has interfered with the normal physiology in a negative manner [29]. In the case of ‘HoneySweet’, the transgene cassette includes an expressed *NPTII* gene, an expressed *UidA* gene, and low level of RNA expression of the PPV coat protein gene without any detectable protein [4,7]. These added genes have no predicted effect on the normal physiology of the fruit. A previous study using sampling in one year and at one location demonstrated that fruit composition of ‘HoneySweet’ fell within a range of other plum cultivars, particularly similar to its maternal parent ‘Bluebyrd’ [6]. To further verify this conclusion, this expanded study was performed to include ‘HoneySweet’ grown at three different locations (US, CZ, ESs) as well as fruit from three different years at one location (US). The comparators were also grown at three or more locations and years as well as many commercial cultivars at numerous locations in Europe and CA. The current study illustrated the wide range of values obtained from both ‘HoneySweet’ and numerous comparators. Consistent with the previous study, the various components measured in ‘HoneySweet’ were in the range of commercial plums. Effects of location as well as year (Fig 3, S1 Fig and Table 3) were evident, not just in ‘HoneySweet’ but also in the two replicated non-transgenic cultivars, ‘Stanley’ and’ Jojo’. In only one component, titratable acids, did ‘HoneySweet’ differ from the majority of the other samples, regardless of location or year, although ‘Haganta’ from DE as well as several of the ‘Jojo’ samples had titratable acids in the same range. The previous study also showed that the maternal parent, ‘Bluebyrd’, had higher titratable acids [6] suggesting that this was not due to the presence of transgenes but a genetically different background. The interplay of acids and sugar on the consumers perception of sweetness is complex and high acidity can be offset by various sugar levels, such that high acid fruit cultivars are acceptable in the market [30–31].

Overall, the fruit composition of ‘HoneySweet’ falls within the range determined for a wide variety of plums grown in diverse locations. There was variation within each cultivar by location and by harvest year while very little variation was detected in duplicate samples or in sampling the same tree at different harvest dates. This variation by location or year is not unexpected and has been previously reported for various commodities including plums [32–36]. It points out that using composition comparisons to look for abnormal deviations potentially caused by transgenes is not simple, as the deviations can be quite large that are caused by differences in location and/or year. Those location/year differences could be due to differences in cultural practices, environmental factors, fruit load, and handling of the fruit samples.

The results presented here demonstrate that the composition values vary by location and by year in the same tree more than they do by different trees in the same location or by cultivars. The data also suggested that the variation in composition is driven as much by environment as it is by cultivar differences. Again, this is not an unexpected result. It draws into question the applicability of this broad sweeping analysis for risk-assessment purposes in the absence of a specific hypothesis or metabolic target- for example if the genetic modification was intended to alter amino acid metabolism [37].

Similar stages of maturity is obliquely addressed in this study, that is the dependency of composition results on the developmental stage. Acids and moisture tend to decrease, sugars increase and color development change as ripening proceeds [38]. Even at an optimum harvest date individual fruit collected at the same time vary in maturity based on firmness [39]. This is why we batched fruit and ground them to get a uniform sample that would represent the average values. In addition, when we sampled one week apart from the same tree, we saw relatively few differences, suggesting that fully mature fruit were chosen at each sampling time. The potentially significant differences that a composition analyses would yield due to differences in maturity between samples again makes interpretation of data difficult, especially to determine if unpredicted results are due to the insertion of a transgene with no predictable phenotypic changes. Certainly, more samples and sampling times could expand the range of values, especially in different regions. The plantings we analyzed covered the range of regions where plums are grown giving at least the range affected by different climates.

One aspect of this broader study was to see what the effect of PPV infection would have on the tolerant cultivar ‘Stanley’ and in a general manner the pressure of PPV on ‘HoneySweet’. A decrease was seen in total carbohydrates in the infected ‘Stanley’ relative to uninfected ‘Stanley’, supporting earlier studies [26–27]. ‘HoneySweet’ grown in Europe, had changes in carotenes sugar and acid suggesting abiotic stress responses to the virus pressure [40]. It is hard to analyze either of these observations as too few trees were sampled to separate the results from a management and location influence.

We did not address the potential for changes in the epistatic control of fruit development and ripening that might occur in ‘HoneySweet’ especially at the sRNA level [41–42]. The changes in carotenes may come through changes in miRNAs such as miR159, miR828 and miR858 that regulate many MYB transcription factors that affect color pathways amongst others in apple [43]. ‘HoneySweet’ is resistant through the continued production of sRNAs related to PPV which may disturb the patterns of key regulatory sRNAs that affect composition. This may be true, but the end result is still that the fruit composition of the transgenic plum cultivar HoneySweet falls within the range of other commercial plum cultivars grown in a wide range of geographical regions and environmental conditions. By this definition ‘HoneySweet’ has the same nutritional properties as other commercial plums and the presence of the transgenes as well as the mechanism of resistance has no appreciable effect.

## Supporting information

S1 Fig Individual fruit values by location. Abbreviations as in Fig 3 legend.
(PDF)

S1 Table Assay protocols. (DOCX)

S2 Table Tests that resulted in <50% of all samples having no detectable amounts. (DOCX)

S3 Table Tests that resulted in no detectable amounts. (DOCX)

S4 Table Comparison of PPV infected ‘Stanley’ and non-infected and PPV pressured ‘HoneySweet’ and non-pressured. (DOCX)
