# Supplementary figures and images for: Multilocation comparison of fruit composition for ‘HoneySweet’, an RNAi based plum pox virus resistant plum
Source: PLoS One. 2019 Mar 22;14(3):e0213993. doi: 10.1371/journal.pone.0213993 (PMC6430400; doi:10.1371/journal.pone.0213993)

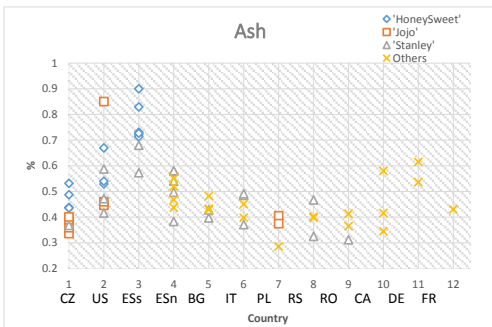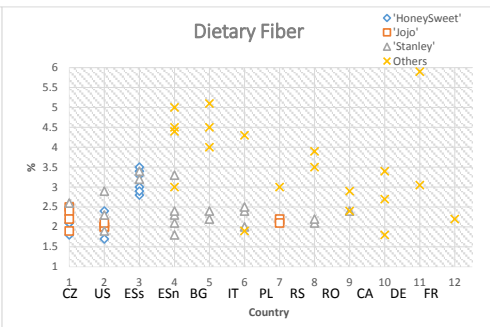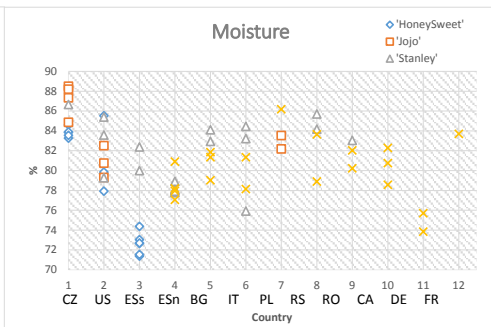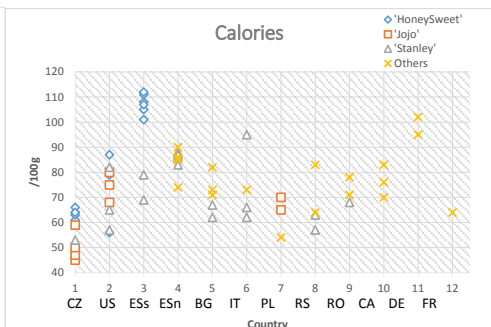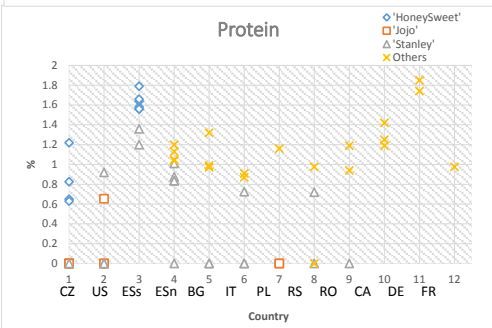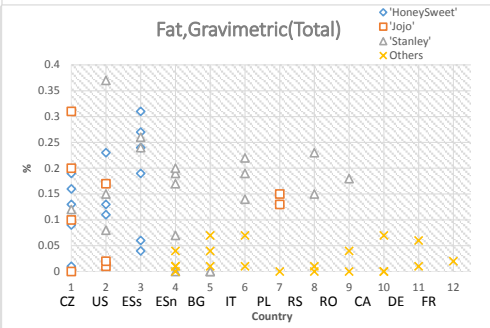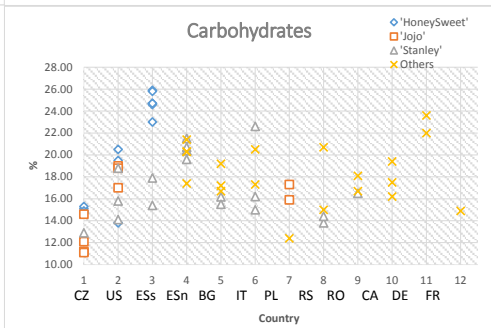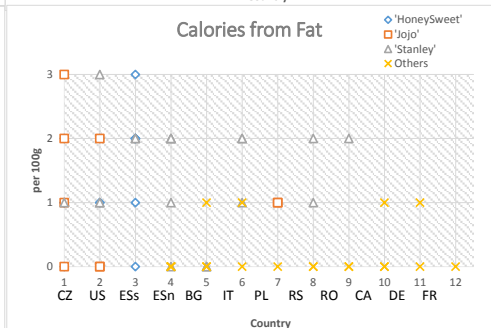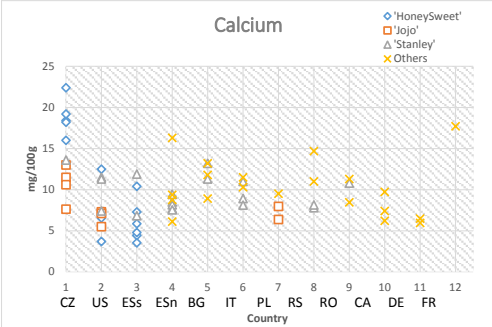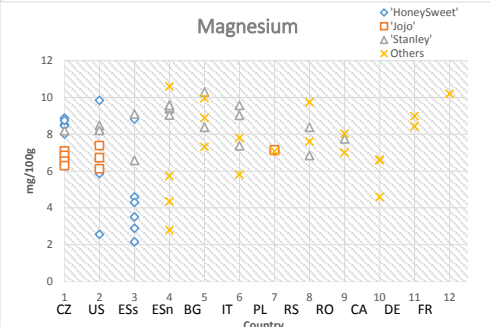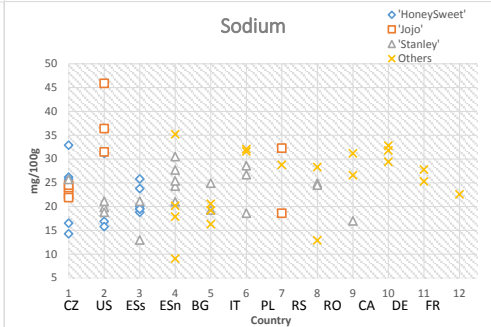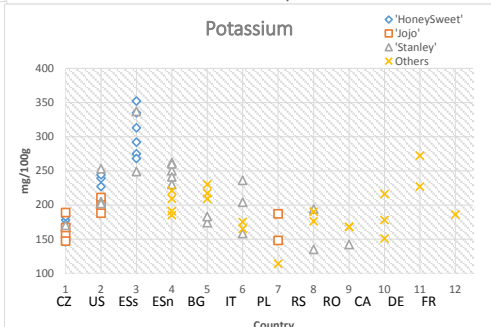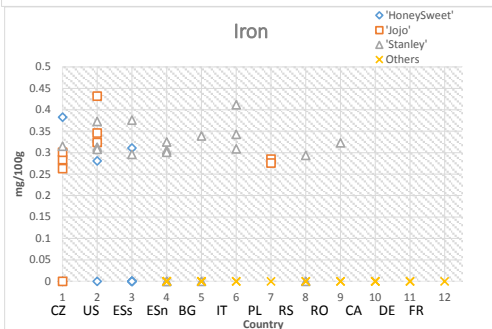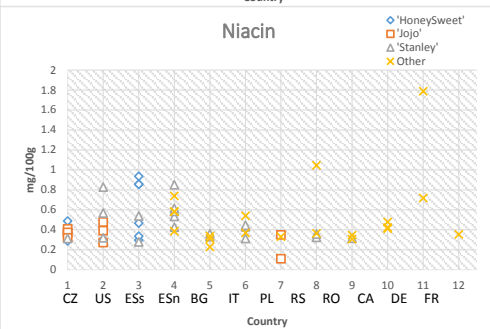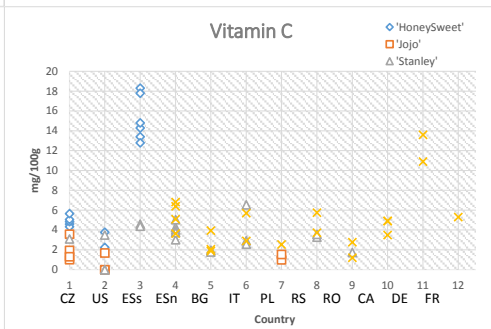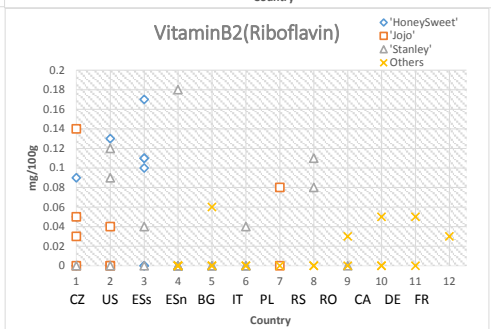

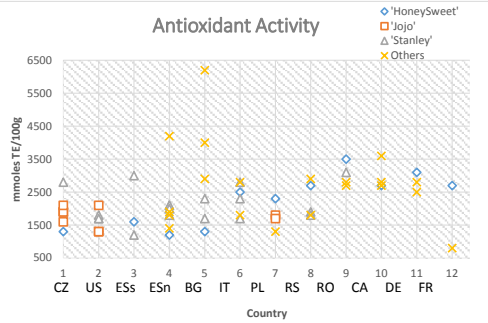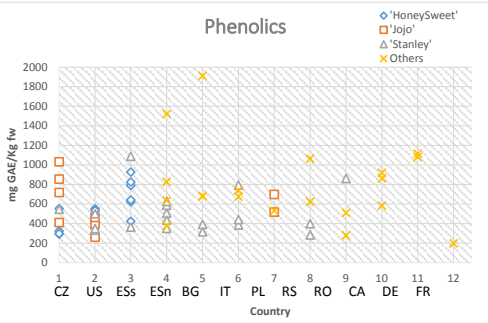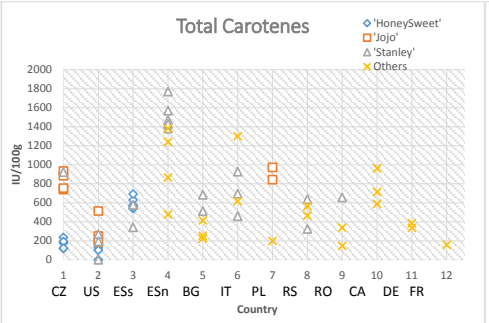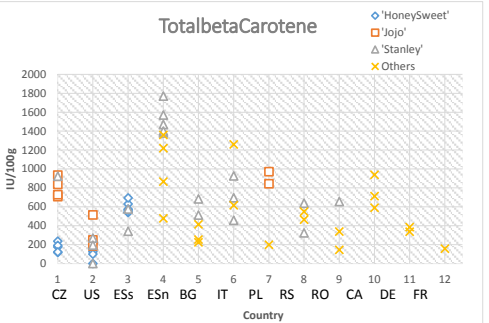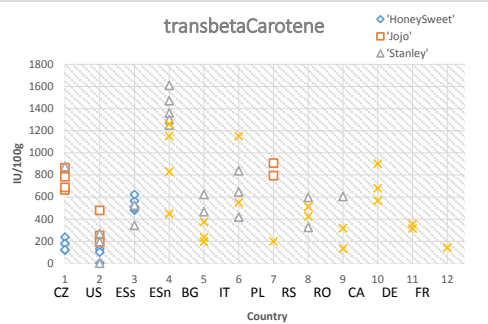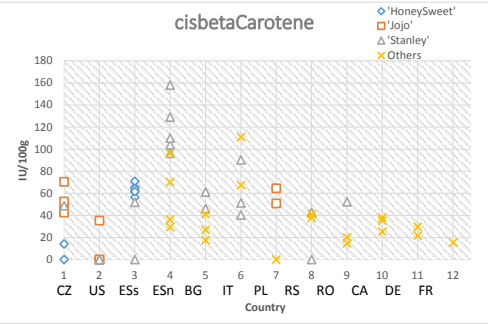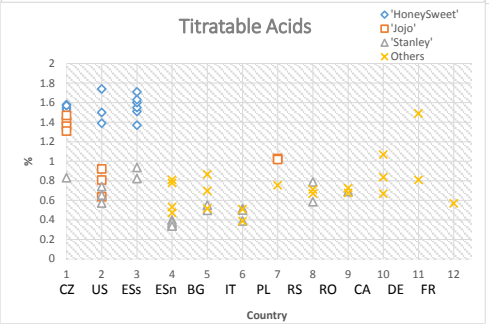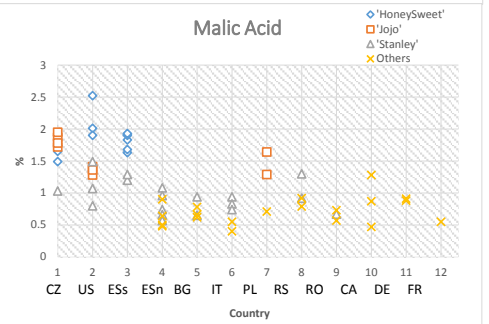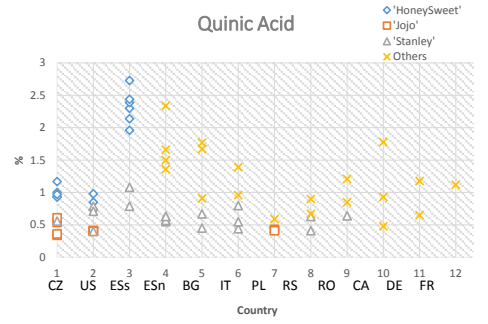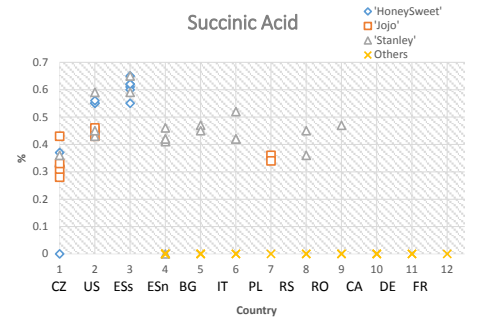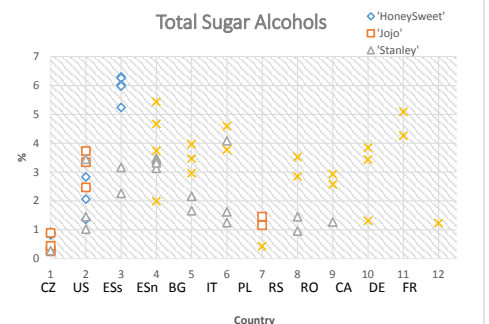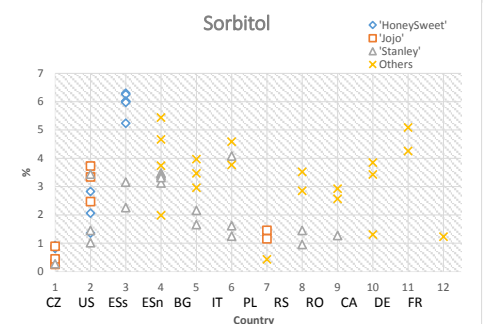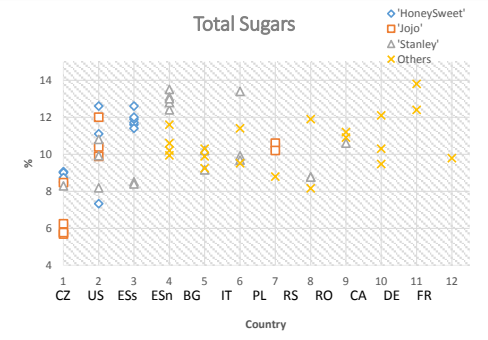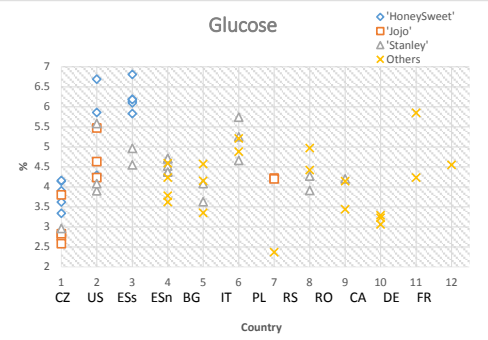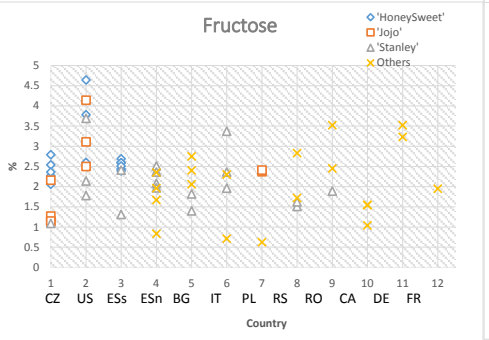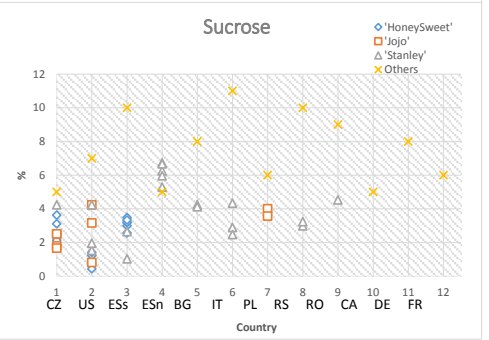

Supplement: S1 Fig — Abbreviations as in Fig 3 legend. (PDF) [file pone.0213993.s001.pdf]
