# Supplementary material for: Multilocation comparison of fruit composition for ‘HoneySweet’, an RNAi based plum pox virus resistant plum
Source: PLoS One. 2019 Mar 22;14(3):e0213993. doi: 10.1371/journal.pone.0213993 (PMC6430400; doi:10.1371/journal.pone.0213993)
Supplement: S1 Table — (DOCX) [file pone.0213993.s002.docx]

| Table S1. Assay Protocols |  |
| --- | --- |
| Assay Name | Assay # |
| AOAC 979.10 and AACC 76-11 | 1 |
| AOAC 2001.13 | 2 |
| AOAC 2011.14 | 3 |
| AOAC 942.15, 962.12, 984.24 | 4 |
| AOAC 986.13 | 5 |
| AOAC: 2005.07* | 6 |
| AOAC: 923.03 | 7 |
| AOAC: 942.23, 981.15, 970.65* | 8 |
| AOAC: 944.13, 960.46 | 9 |
| AOAC: 945.43, 934.01* | 10 |
| AOAC: 948.15, 922.06, 925.32, 950.54, 922.09* | 11 |
| AOAC: 967.22, 984.26* | 12 |
| AOAC: 975.03, 985.01, 990.08 | 13 |
| AOAC: 991.43* | 14 |
| AOAC: 992.15; AACC: 46-30 | 15 |
| Determined through calculation | 16 |
| DPPH Method | 17 |
| Internal HPLC method | 18 |
| Internal Method | 19 |
| * with modifications |  |
