# Supplementary material for: Multilocation comparison of fruit composition for ‘HoneySweet’, an RNAi based plum pox virus resistant plum
Source: PLoS One. 2019 Mar 22;14(3):e0213993. doi: 10.1371/journal.pone.0213993 (PMC6430400; doi:10.1371/journal.pone.0213993)
Supplement: S2 Table — (DOCX) [file pone.0213993.s003.docx]

| Table S2. Tests that resulted in <50% of all samples having No Detectable amounts | | | | | | | |
| --- | --- | --- | --- | --- | --- | --- | --- |
| Test | Assay | Lowest limit Detection | Unit | Stanley | JoJo | HoneySweet | Others |
| VitaminB1 (Thiamine-HCL(US)) | 8 | <0.03 mg/100g | mg/100g | 0.03(0.01) | ND | 0.03(0.01) | ND |
| VitaminB1 (Thiamine (EU)) | 8 | <0.02 mg/100g | mg/100g | 0.02(0.01) | ND | 0.02(0.01) | ND |
| VitaminB2 (Riboflavin) | 8 | <0.03 mg/100g | mg/100g | 0.09(0.05) | 0.07(0.05) | 0.11(0.06) | 0.04(0.02) |
| alphacarotene | 6 | <0.5 IU/100 g | IU/100g | 33.9(7.78) | 34.5(18.3) | ND | 23.9(11.2) |
| Oxalic | 5 | < 0.01 % | % | ND* | 0.01(0) | 0.02(0.01) | 0.02(0.01) |
| Citric | 5 | < 0.01 % | % | 0.07(0.02) | ND | 0.02(0.01) | 0.01(0) |
| Tartaric | 5 | < 0.01 % | % | 0.08(0.04) | 0.07(0.04) | 0.03(0.01) | ND |
| Succinic | 5 | <0.01 % | % | 0.46(0.13) | 0.38(0.07) | 0.51(0.19) | ND |
| Glutaric | 5 | <0.01 % | % | 0.08(0.03) | 0.10(0.05) | 0.13(0.07) | ND |
| Acetic | 5 | < 0.01 % | % | 0.06(0.02) | ND | 0.06(0.03) | ND |
| *ND-amount was below detectable levels of the assay for all samples | | | | | | | |
