# Supplementary material for: Multilocation comparison of fruit composition for ‘HoneySweet’, an RNAi based plum pox virus resistant plum
Source: PLoS One. 2019 Mar 22;14(3):e0213993. doi: 10.1371/journal.pone.0213993 (PMC6430400; doi:10.1371/journal.pone.0213993)
Supplement: S3 Table — (DOCX) [file pone.0213993.s004.docx]

| Table S3. Tests that resulted in No Detectable Amounts | | |  |
| --- | --- | --- | --- |
| Test | Assay | LDL | Unit |
| TotalStarch | 1 | <1% | % |
| Lactic | 5 | <0.01% | % |
| Fumaric | 5 | <0.01% | % |
| Vitamin A (Retinol) | 2 | <50 | IU/100g |
| Erythritol | 19 | <0.1% | % |
| Xylitol | 19 | <0.1% | % |
| Lactitol | 19 | <0.1% | % |
| Maltitol | 19 | <0.1% | % |
| Galactose | 19 | <0.1% | % |
| Isomalt | 19 | <0.1% | % |
| Lactose | 19 | <0.1% | % |
| Maltose | 19 | <0.1% | % |
| Mannitol | 19 | <0.1% | % |
| Trehalose | 19 | <0.1% | % |
