# Supplementary material for: Multilocation comparison of fruit composition for ‘HoneySweet’, an RNAi based plum pox virus resistant plum
Source: PLoS One. 2019 Mar 22;14(3):e0213993. doi: 10.1371/journal.pone.0213993 (PMC6430400; doi:10.1371/journal.pone.0213993)
Supplement: S4 Table — (DOCX) [file pone.0213993.s005.docx]

| **S4 Table.** Influence of infection of pressure of PPV on 'Stanley' and 'HoneySweet' fruit. | | | | | | | |  |  |
| --- | --- | --- | --- | --- | --- | --- | --- | --- | --- |
| Tree | Country | Carbohydrates | transbeta carotene | Total Carotene | Malic | Sucrose | Fructose | Total Sugars | VitaminC |
|  |  | % | IU/100g | IU/100g | % | % | % | % | mg/100g |
| **'Stanley'** |  |  |  |  |  |  |  |  |  |
| ANOVA ρ-value | | 0.028 | 0.275 | 0.278 | 0.480 | 0.423 | 0.064 | 0.021 | 0.932 |
| PPV infected |  |  |  |  |  |  |  |  |  |
| 1 | BG | 16.17 | 623 | 684 | 0.65 | 4.25 | 1.82 | 10.2 | 1.97 |
| 2 | BS | 15.5 | 466 | 512 | 0.94 | 4.12 | 1.4 | 9.15 | 1.78 |
| 1.5 | Ess | 17.9 | 525 | 577 | 1.2 | 2.65 | 1.31 | 8.51 | 4.6 |
| 2.3 | Ess | 15.4 | 342 | 342 | 1.29 | 1.03 | 2.41 | 8.4 | 4.38 |
| Uninfected |  |  |  |  |  |  |  |  |  |
| T2 | Esn | 21.5 | 1610 | 1770 | 1.08 | 6.73 | 2.08 | 13.5 | 4.27 |
| T 3 | ESn | 19.6 | 1470 | 1570 | 0.74 | 5.29 | 2.37 | 12.4 | 3.02 |
| T1 | ESn | 20.4 | 1360 | 1470 | 0.66 | 6.25 | 2.51 | 13.1 | 4.01 |
| T1 (duplicate) | ESn | 20.4 | 1250 | 1380 | 0.96 | 5.96 | 2.36 | 12.8 | 3.76 |
| T2(duplicate) | ESn | 20.7 | 1300 | 1430 | 0.57 | 6.67 | 1.97 | 13 | 5.04 |
| 2010 | US | 20.5 | ND | ND | 2.52 | 1.23 | 4.64 | 12.6 | ND |
| 2008 | US | 19.5 | 127 | 127 | 2.01 | 1.42 | 3.78 | 11.1 | 2.24 |
| 2011 | US | 13.8 | 101 | 101 | 1.9 | 0.44 | 2.6 | 7.33 | 3.78 |
| Tree | Country | Carbohydrates | transbetacarotene | Total Carotene | Malic | Sucrose | Fructose | Total Sugars | VitaminC |
| **'HoneySweet'** | |  |  |  |  |  |  |  |  |
| ANOVA ρ-value | | 0.393 | 0.018 | 0.021 | 0.014 | 0.000 | 0.003 | 0.807 | 0.023 |
| PPV pressure |  |  |  |  |  |  |  |  |  |
| V/2 | CZ | 15.1 | 236 | 236 | 1.65 | 2.13 | 2.79 | 9.06 | 4.6 |
| V-2(Duplicate) | CZ | 14.7 | 122 | 122 | 1.49 | 2.48 | 2.36 | 8.74 | 5.66 |
| V/5 | CZ | 14.8 | 177 | 177 | 1.9 | 1.8 | 2.54 | 8.5 | 4.33 |
| V/7 | CZ | 14.9 | 178 | 192 | 1.74 | 3.11 | 2.27 | 9 | 5.06 |
| 5.3 7/21 | ESs | 23 | 563 | 629 | 1.83 | 3 | 2.44 | 11.6 | 13.4 |
| 5.3 7/25 | ESs | 24.6 | 622 | 693 | 1.63 | 3.48 | 2.39 | 11.7 | 12.8 |
| 5.3 7/25 | ESs | 24.7 | 481 | 543 | 1.9 | 2.57 | 2.69 | 11.4 | 14.3 |
| 7.3 7/21 | ESs | 25.9 | 511 | 575 | 1.68 | 3.18 | 2.59 | 12.6 | 18.3 |
| 7.3 7/25 | ESs | 24.7 | 483 | 540 | 1.92 | 3.2 | 2.59 | 11.9 | 14.8 |
| 7.3 7/25 | ESs | 25.8 | 502 | 564 | 1.93 | 3.33 | 2.51 | 12 | 17.8 |
| No PPV |  |  |  |  |  |  |  |  |  |
| 2008 | US | 20.5 | ND | ND | 2.52 | 1.23 | 4.64 | 12.6 | ND |
| 2010 | US | 19.5 | 127 | 127 | 2.01 | 1.42 | 3.78 | 11.1 | 2.24 |
| 2011 | US | 13.8 | 101 | 101 | 1.9 | 0.44 | 2.6 | 7.33 | 3.78 |
